# Supplementary material for: CoHIT: a one-pot ultrasensitive ERA-CRISPR system for detecting multiple same-site indels
Source: Nat Commun. 2024 Jun 12;15:5014. doi: 10.1038/s41467-024-49414-7 (PMC11169540; doi:10.1038/s41467-024-49414-7)
Supplement: Supplementary file 1 — Supplementary information [file 41467_2024_49414_MOESM1_ESM.pdf]

Supplementary information for

CoHIT: A one-pot ultrasensitive ERA-CRISPR system for detecting multiple same-site indels

Yin Liu,[1] Xinyi Liu,[1] Dongyi Wei, Lu Dang, Xiaoran Xu, Shisheng Huang, Liwen Li, Sanyun Wu, Jinxian Wu, Xiaoyan Liu, Wenjun Sun, Wanyu Tao, Yongchang Wei, Xingxu Huang, Kui Li,* Xinjie Wang,* Fuling Zhou *

[1] Yin Liu and Xinyi Liu contributed equally to this research
* Corresponding authors. Email: zhoufuling@whu.edu.cn(F.Z.); wang_xin_jie@126.com(X.W.); likui@caas.cn(K.L.);

This PDF file includes:

Supplementary Figure 1 to 26
Supplementary Table 1

Table of Contents

Supplementary Figure 1 | Mismatch tolerance comparison assay of WT AsCas12a and LbCas12a

Supplementary Figure 2 | PAM identification of Cas12a variants

Supplementary Figure 3 | Comparison between CCTG-crRNA1 and TCTG-crRNA1 to detect top six *NPM1* c.863_864 4-bp insertions

Supplementary Figure 4 | Comparison of six AsCas12a variants on mismatch tolerance

Supplementary Figure 5 | Optimization of the CoHIT system on buffers

Supplementary Figure 6 | Time-course of fluorescence value changes of the ERA-CRISPR one-pot assay at different reaction temperatures

Supplementary Figure 7 | Concentration screen of FAM-ssDNA-BHQ1 reporter, enAsU-R Cas12a protein, and crRNA for CoHIT system

Supplementary Figure 8 | LoD assay of the CoHIT system using plasmid templates

Supplementary Figure 9 | LoD assay of the CoHIT system using genomic DNA templates from AML patients

Supplementary Figure 10 | Screen crRNAs and ERA primers for the *KIT* gene p.W557_K558del detection

Supplementary Figure 11 | Screen crRNAs and ERA primers for the *BRAF* gene p.V487_T491del detection

Supplementary Figure 12 | Screen crRNAs and ERA primer for the *EGFR* gene p.E746_A750del detection

Supplementary Figure 13 | Detect 2-bp and 1-bp indels using the CoHIT system

Supplementary Figure 14 | Template input assay of the CoHIT system

Supplementary Figure 15 | Naked-eye results of CoHIT detection of the *NPM1* gene c.863_864 4-bp insertion status of 108 AML patient samples

Supplementary Figure 16 | FGS, NGS, and CoHIT detection results of the *NPM1* c.863_864 4-bp insertion status of AML patient samples (P1 ~ 20)

Supplementary Figure 17 | FGS, NGS, and CoHIT detection results of the *NPM1* c.863_864 4-bp insertion status of AML patient samples (P21 ~ 40)

Supplementary Figure 18 | FGS, NGS, and CoHIT detection results of the *NPM1* c.863_864 4-bp insertion status of AML patient samples (P41 ~ 60)

Supplementary Figure 19 | FGS, NGS, and CoHIT detection results of the *NPM1* c.863_864 4-bp insertion status of AML patient samples (P61 ~ 80)

Supplementary Figure 20 | FGS, NGS, and CoHIT detection results of the *NPM1* c.863_864 4-bp insertion status of AML patient samples (P81 ~ 100)

Supplementary Figure 21 | FGS, NGS, and CoHIT detection results of the *NPM1* c.863_864 4-bp insertion status of AML patient samples (P101 ~ 108)

Supplementary Figure 22 | FGS chromatograms of the eight bone marrow samples of Patient-MRD-1, collected between 10/2021 (First visit) and 01/2023

Supplementary Figure 23 | FGS, NGS, and CoHIT detection results of Patient-MRD-2

Supplementary Figure 24 | FGS, NGS, and CoHIT detection results of Patient-MRD-3

Supplementary Figure 25 | Gene mutation sites and types of targets 1 ~ 7 in the microfluidic chip-based multiplexing CoHIT assay

Supplementary Figure 26 | Off-target evaluation of Cas12a proteins on four sites

Supplementary Table 1 | Comparison between different genotyping methods

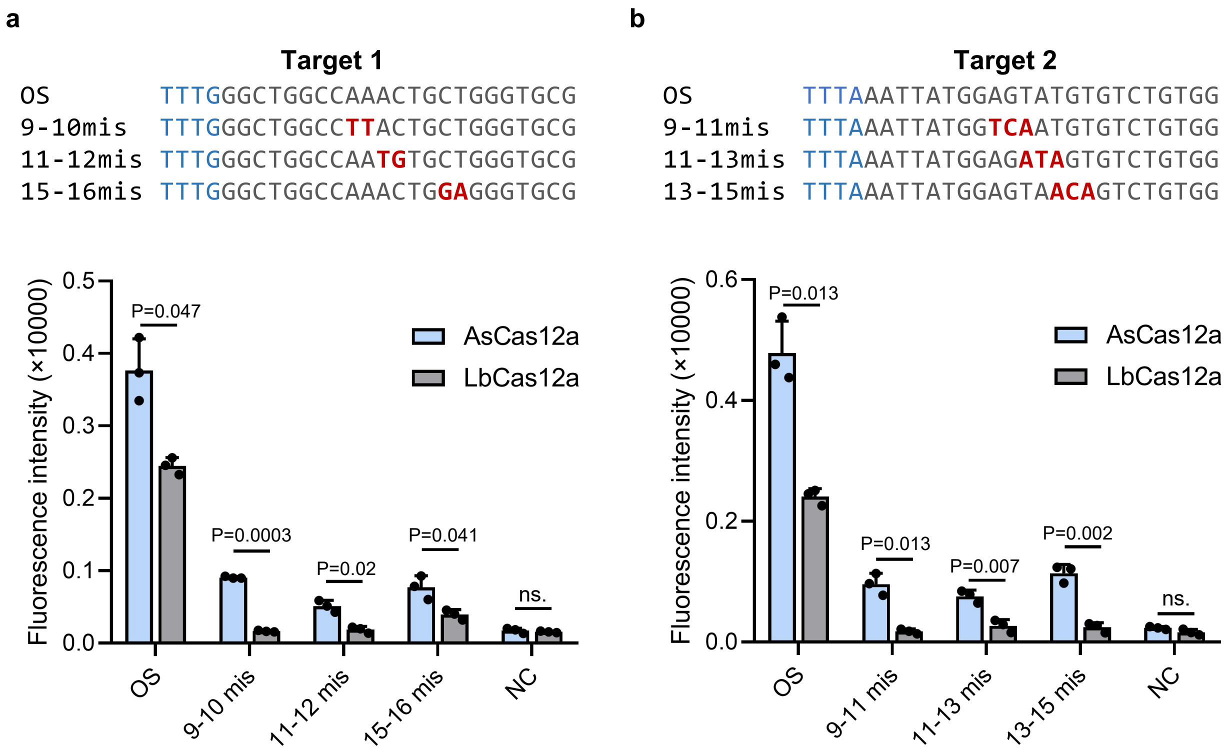

Supplementary Figure 1 | Mismatch tolerance comparison assay of WT AsCas12a and LbCas12a. **a** The Original sequence (OS, completely complementary to the crRNA) of Target 1 and three seuquences with two-base mismatches at different locations are shown above. The PAM and mismatch bases are marked in blue and red, respectively. 2E10 copies of these DNA substrates are directly detected using Cas12a-induced in vitro cleavage assay. The fluorescence intensity of 30 min of reaction is shown below. Values and error bars reflect the means and standard deviation (s.d.) of three biological replicates. P values are determined by two-tailed Student's t-tests, ns., no significance. **b** The OS, three-base mismatch sequences, and fluorescence result of Target 2. Source data are provided as a Source Data file. Values and error bars reflect the means and standard deviation (s.d.) of three biological replicates. P values are determined by two-tailed Student's t-tests, ns., no significance.

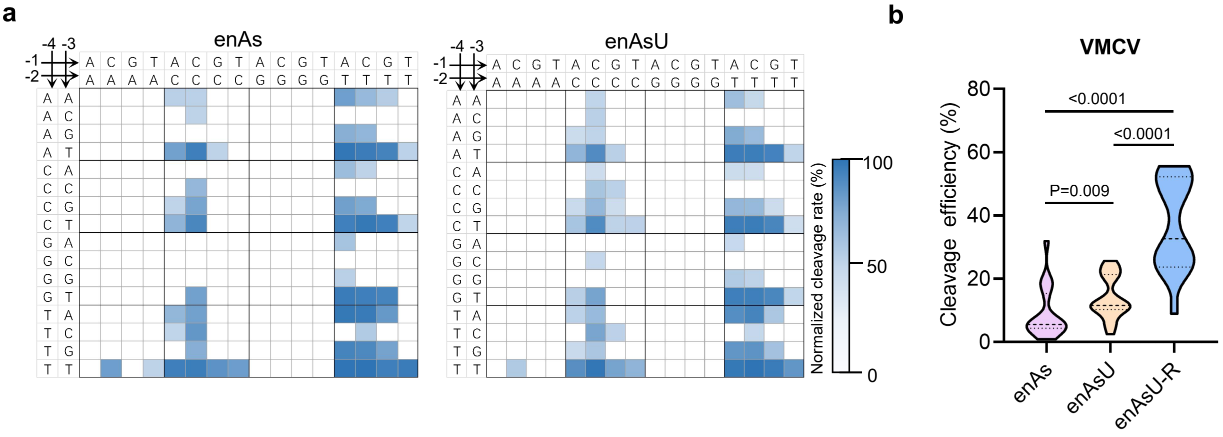

Supplementary Figure 2 | PAM identification of Cas12a variants. a The normalized cleavage rates of enAs and enAsU Cas12a variants for synthesized substrates with all possible 4-base PAMs. The intensity of blue color represents the cleavage activity of Cas12a nuclease. **b** Cleavage efficiency comparison of enAs, enAsU, and enAsU-R on VMCV PAM (n=18 targets). P values are determined by two-tailed Student's t-tests. Source data are provided as a Source Data file.

a

Insertion type	CCTG-crRNA1	TCTG-crRNA1
TCTG	1	0
CATG	1	2
CCTG	0	1
CCAG	1	2
CCGG	1	2
CTTG	1	2

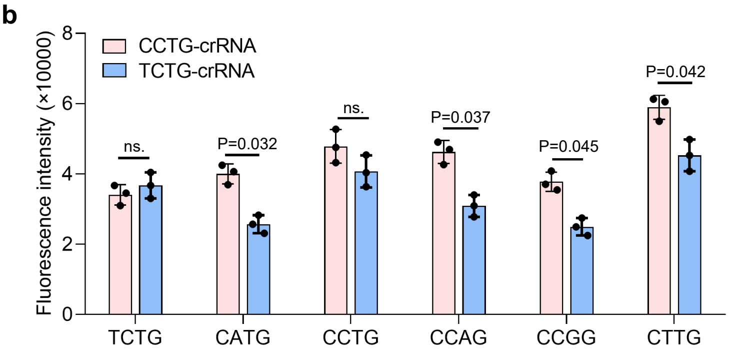

Supplementary Figure 3 | Comparison between CCTG-crRNA1 and TCTG-crRNA1 to detect top six *NPM1* c.863_864 4-bp insertions. **a** Numbers of mismatch bases between the two crRNAs and the top six insertions. **b** Statistical chart of fluorescence values of enAsU-R-induced in vitro cleavage assay with the two crRNAs, using 2E10 copies of DNA fragments of the top six insertions as substrates, reacting for 15 min at 37°C. Values and error bars reflect the means and s.d. of three biological replicates. P values are determined by two-tailed Student's t-tests, ns., no significance. Source data are provided as a Source Data file.

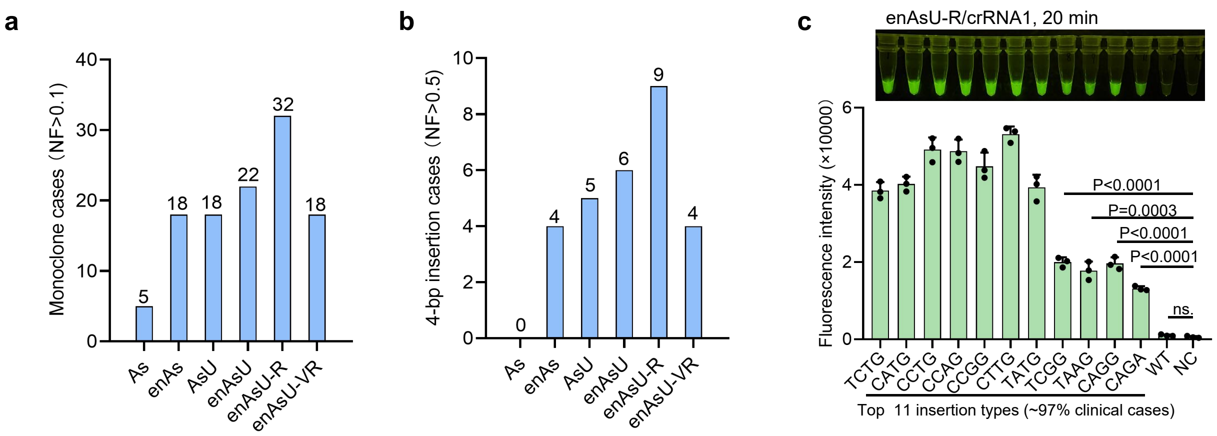

Supplementary Figure 4 | Comparison of six AsCas12a variants on mismatch tolerance. **a** Detection of 32 random TA clones carrying the *NPM1* c.863_864insNNNN mutated fragments by different Cas12a-induced in vitro cleavage assays. The histogram shows the number of monoclone cases with NF > 0.1 for each Cas12a protein variant. NF, normalized fluorescence. **b** Detection of the top eleven c.863_864insNNNN mutations by different Cas12a-induced in vitro cleavage assays. The histogram shows the number of 4-bp insertion cases with NF > 0.5 for each Cas12a protein variant. **c** Final fluorescence values of the top eleven c.863_864insNNNN mutations by enAsU-R/crRNA1 detection. The fluorescence image shows the naked-eye result under blue light. Values and error bars reflect the means and s.d. of three biological replicates. P values are determined by two-tailed Student's t-tests, ns., no significance. Source data are provided as a Source Data file.

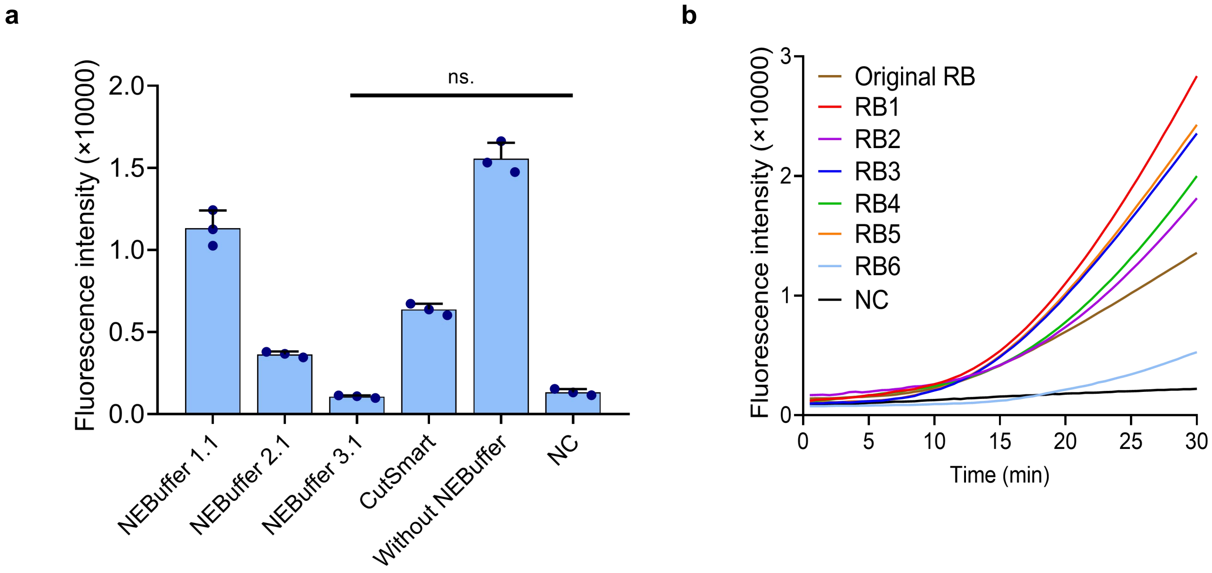

Supplementary Figure 5 | Optimization of the CoHIT system on buffers. a Comparison of a series of NEBuffers for ERA-CRISPR one-pot assay, using 1E6 copies of c.863_864insTCTG plasmid as a template, reacting for 30 min at 37℃. Values and error bars reflect the means and s.d. of three biological replicates. P values are determined by two-tailed Student's t-tests, ns., no significance. **b** Comparison of a series of ERA Reaction Buffers for one-pot assay. Source data are provided as a Source Data file.

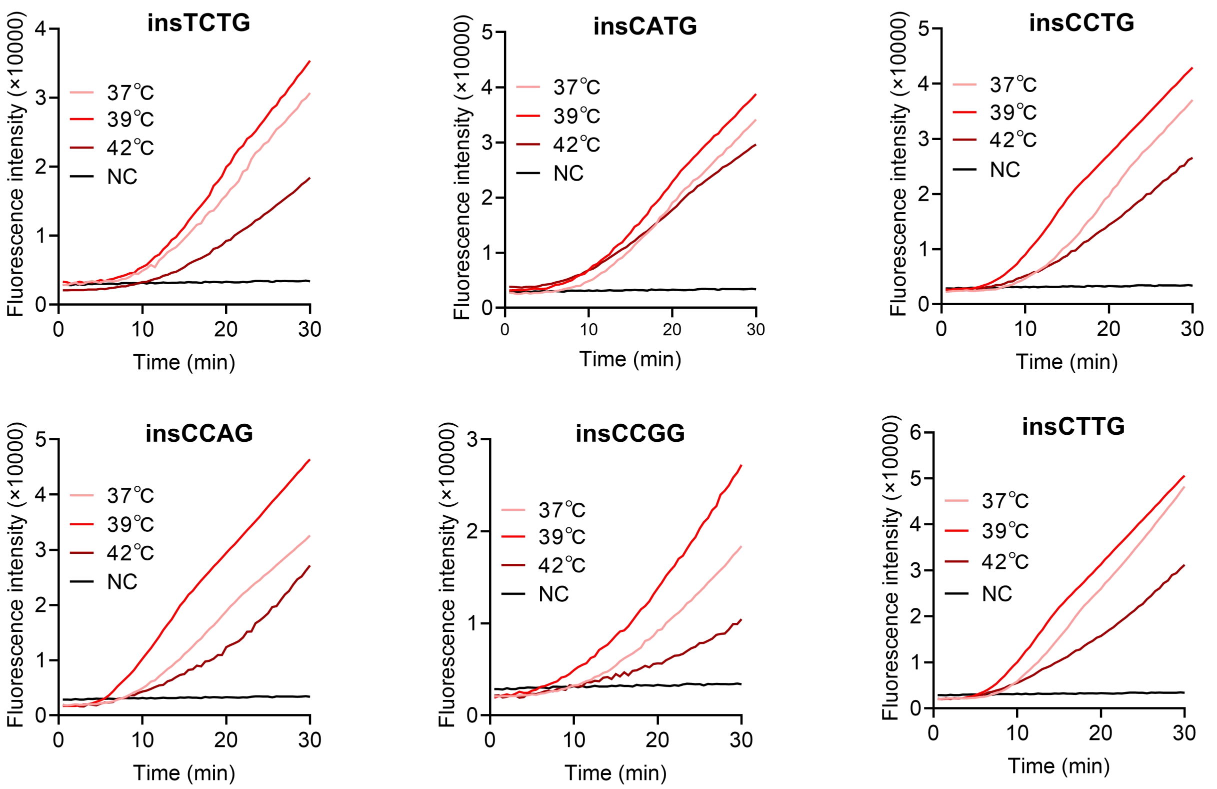

Supplementary Figure 6 | Time-course of fluorescence value changes of the ERA-CRISPR one-pot assay at different reaction temperatures, using 1E6 copies of plasmids carrying the *NPM1* gene c.863_864insTCTG, CATG, CCTG, CCAG, CCGG, and CTTG fragments as the templates, respectively. Source data are provided as a Source Data file.

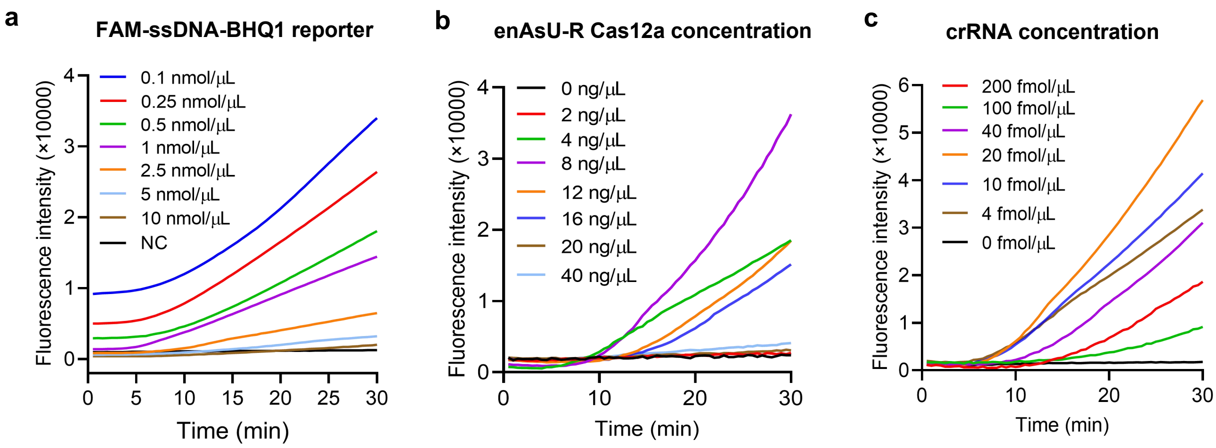

Supplementary Figure 7 | Concentration screen of FAM-ssDNA-BHQ1 reporter (**a**), enAsU-R Cas12a protein (**b**), and crRNA (**c**) for CoHIT system, using 1E6 copies of c.863_864insTCTG plasmid as a template, reacting for 30 min at 39°C. Source data are provided as a Source Data file.

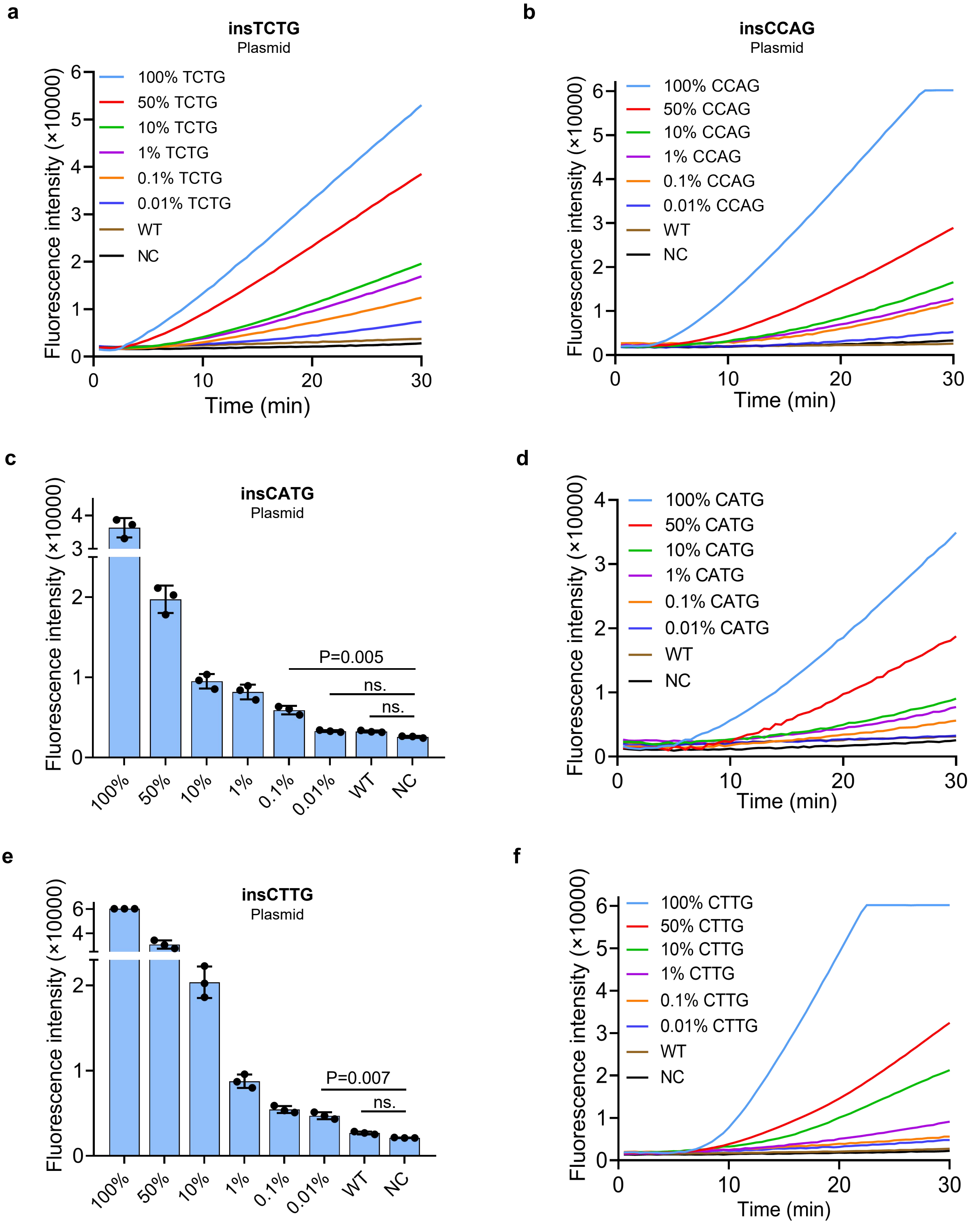

Supplementary Figure 8 | LoD assay of the CoHIT system using plasmid templates. **a** Time-course of the CoHIT detection of the *NPM1* c.863_864insTCTG mutation, using 1E6 copies of plasmid as a template, reacting for 30 min at 39℃. **b** The ime-course for the c.863_864insCCAG mutation. **c-d** The histogram of the final fluorescence intensity and the time-course for c.863_864insCATG mutation detection. Values and error bars reflect the means and s.d. of three biological replicates. P values are determined by two-tailed Student's t-tests, ns., no significance. **e-f** The histogram and the time-course for the c.863_864insCTTG mutation detection. Values and error bars reflect the means and s.d. of three biological replicates. P values are determined by two-tailed Student's t-tests, ns., no significance. Source data are provided as a Source Data file.

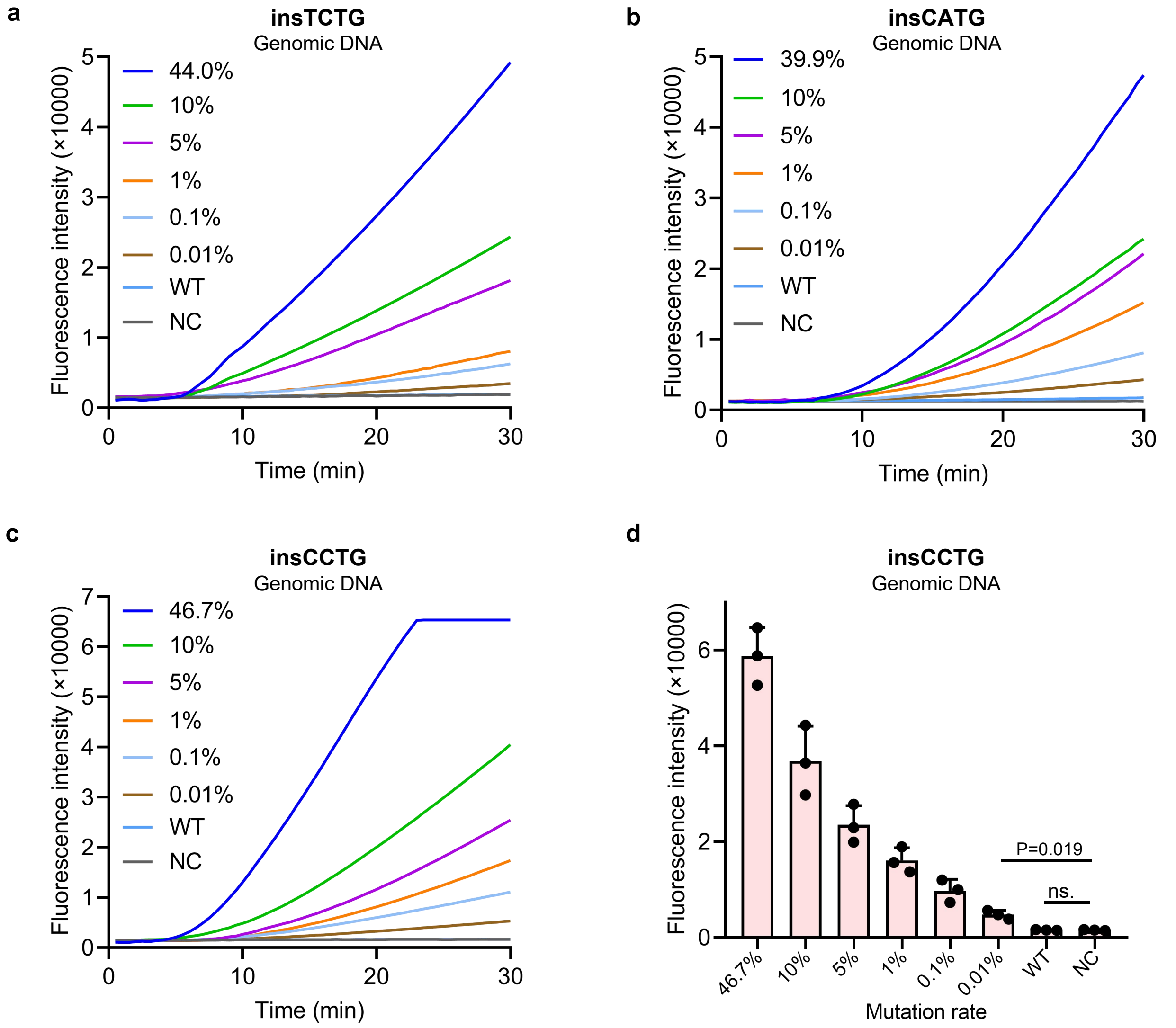

Supplementary Figure 9 | LoD assay of the CoHIT system using genomic DNA templates from AML patients. **a** The time-course of CoHIT detection of the *NPM1* c.863_864insTCTG mutation, with gradient mutation ratios by mixing patient genomic DNA with healthy WT control. **b** The time-course of CoHIT detection of the insCATG mutation. **c** The time-course of CoHIT detection of the insCCTG mutation. **d** Final fluorescence statistics of figure c. Values and error bars reflect the means and s.d. of three biological replicates. P values are determined by two-tailed Student's t-tests, ns., no significance. Source data are provided as a Source Data file.

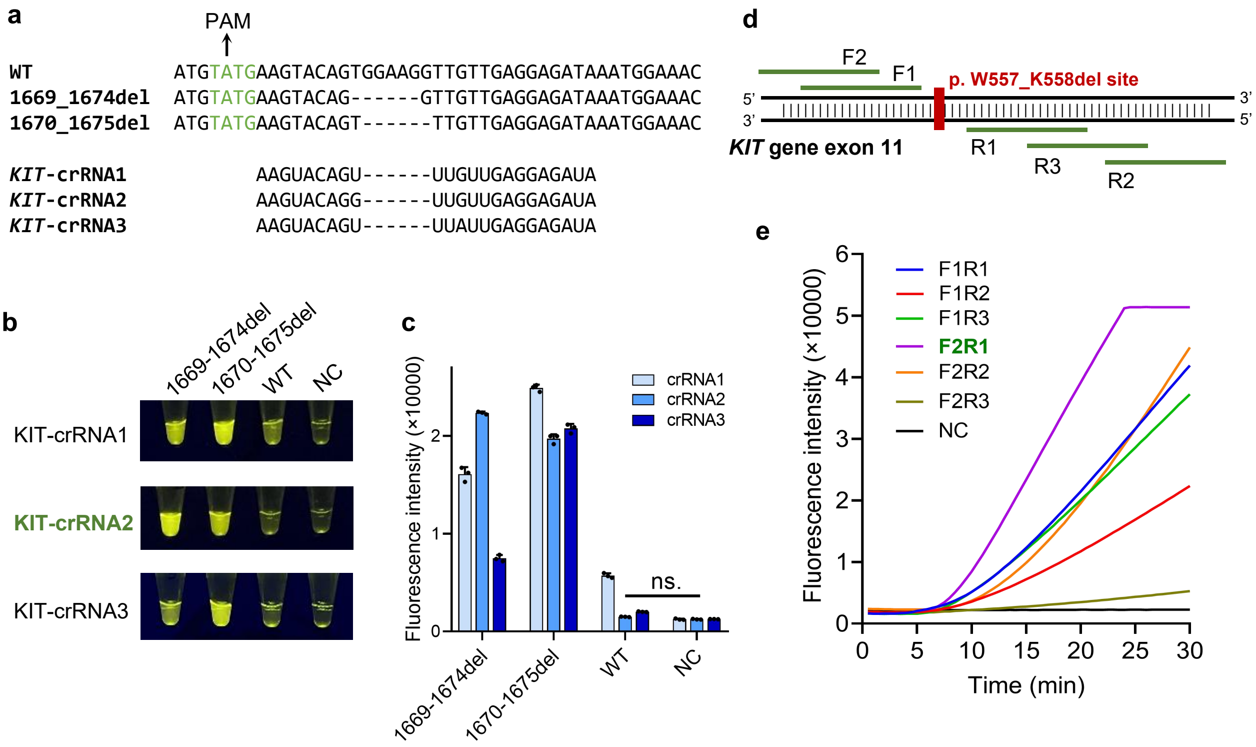

Supplementary Figure 10 | Screen crRNAs and ERA primers for the *KIT* gene p.W557_K558del detection. **a** The upper part shows DNA sequences of the WT and two most common variants (c.1669_1674del and c.1670_1675del) of the *KIT* gene p.W557_K558del mutation, the PAM is marked in green. The lower part shows the RNA sequences of three designed crRNAs for variant detection. **b-c** Sensitivity and specificity assay of *KIT*-crRNA1, 2, and 3, using 2E10 copies of WT and variant DNA fragments as substrates for the enAsU-R in vitro cleavage reaction, reacting for 30 min at 39°C. The naked-eye result and statistical diagram show that crRNA2 has both good sensitivity and specificity for *KIT* gene p.W557_K558del detection. Values and error bars reflect the means and s.d. of three biological replicates. P values are determined by two-tailed Student's t-tests, ns., no significance. **d** Genomic locations of designed forward and reverse ERA primers for the *KIT* target. **e** ERA primer screen for CoHIT detection of the *KIT* mutation, using 1E5 copies of the *KIT* gene c.1669_1674del plasmid as the template, reacting for 30 min at 39°C. The time course shows that F2R1 primer pair has the best performance. Source data are provided as a Source Data file.

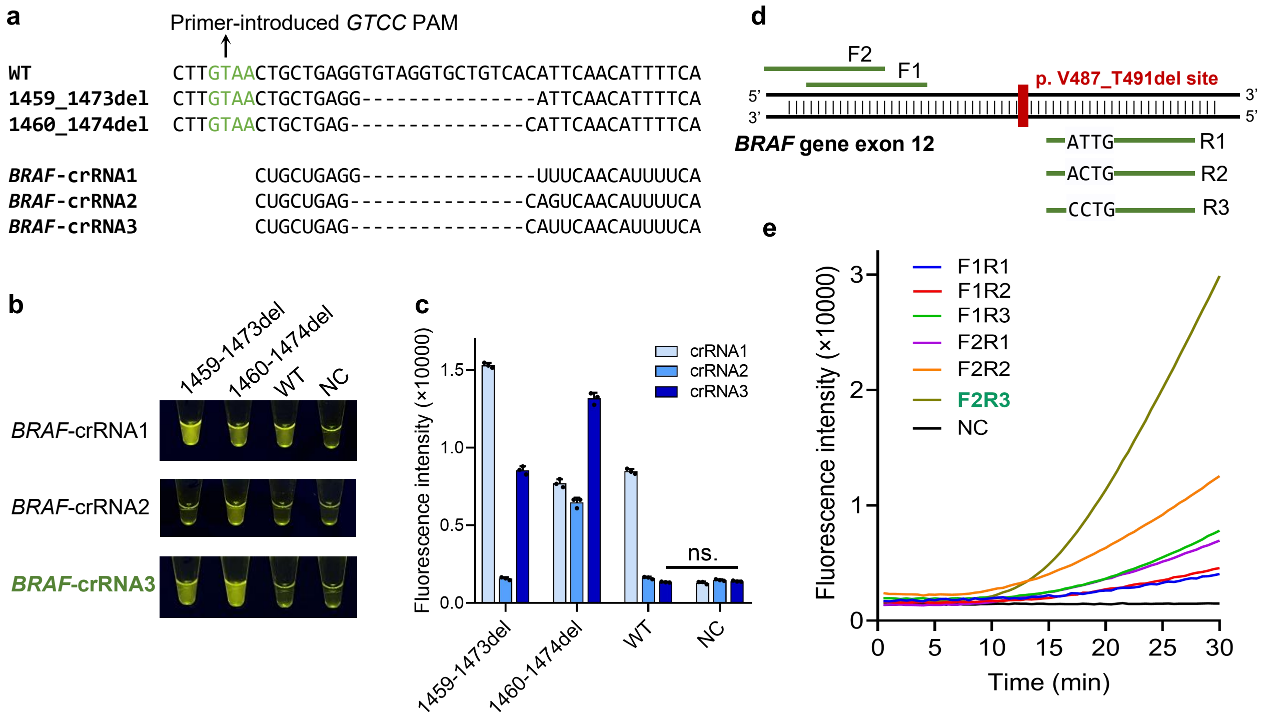

Supplementary Figure 11 | Screen crRNAs and ERA primers for the *BRAF* gene p.V487_T491del detection. **a** The upper part shows the DNA sequences of the WT and two most common variants (c.1459_1473del and c.1460_1474del) of the *BRAF* gene p.V487_T491del mutation, the PAM is marked in green. The lower part shows the the RNA sequences of three designed crRNAs for variant detection. **b-c** Sensitivity and specificity assay of *BRAF*-crRNA1, 2, and 3, using 2E10 copies of WT and variant DNA fragments as substrates for the enAsU-R in vitro cleavage reaction, reacting for 30 min at 39℃. The naked-eye result and statistical diagram show that crRNA3 has both good sensitivity and specificity for the *BRAF* gene p.V487_T491del detection. Values and error bars reflect the means and s.d. of three biological replicates. P values are determined by two-tailed Student's t-tests, ns., no significance. **d** Genomic locations of designed forward and reverse ERA primers for the *BRAF* target, sequences in the reverse primers are artificaially introduced PAMs. **e** ERA primer screen for CoHIT detection of the *BRAF* mutation, using 1E5 copies of the *BRAF* gene c.1459_1473del plasmid as the template, reacting for 30 min at 39℃. The time-course shows that F2R3 primer pair has the best performance. Source data are provided as a Source Data file.

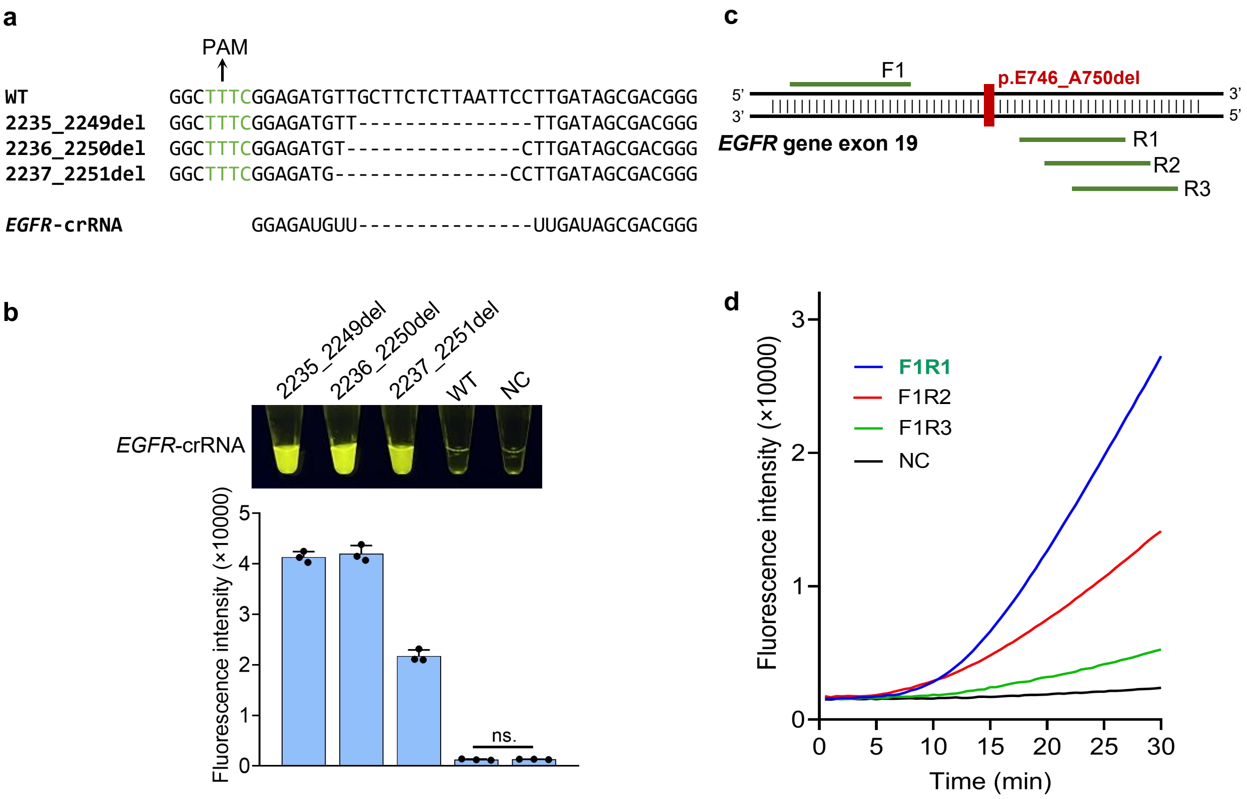

Supplementary Figure 12 | Screen crRNAs and ERA primer for the *EGFR* gene p.E746_A750del detection. **a** The upper part shows the DNA sequences of WT and three most common p.E746_A750del variants (c.2235_2249del, c.2236_2250del and c.2237_2251del) of the *EGFR* gene, the PAM is marked in green. The lower part shows the crRNA sequence for variant detection. **b** Sensitivity and specificity identification of *EGFR*-crRNA, using 2E10 copies of WT and variant DNA fragments as substrates for the enAsU-R in vitro cleavage reaction, reacting for 30 min at 39℃. The naked-eye result and statistical diagram show that the *EGFR*-crRNA has both good sensitivity and specificity for the *EGFR* gene p.E746_A750del detection. **c** Genomic locations of designed forward and reverse ERA primers for the *EGFR* target. Values and error bars reflect the means and s.d. of three biological replicates. P values are determined by two-tailed Student's t-tests, ns., no significance. **d** ERA primer screen for CoHIT detection of the *EGFR* mutation, using 1E5 copies of the *EGFR* gene c.2235_2249del plasmid as the template, reacting for 30 min at 39℃. The time-course shows that F1R1 primer pair has the best performance. Source data are provided as a Source Data file.

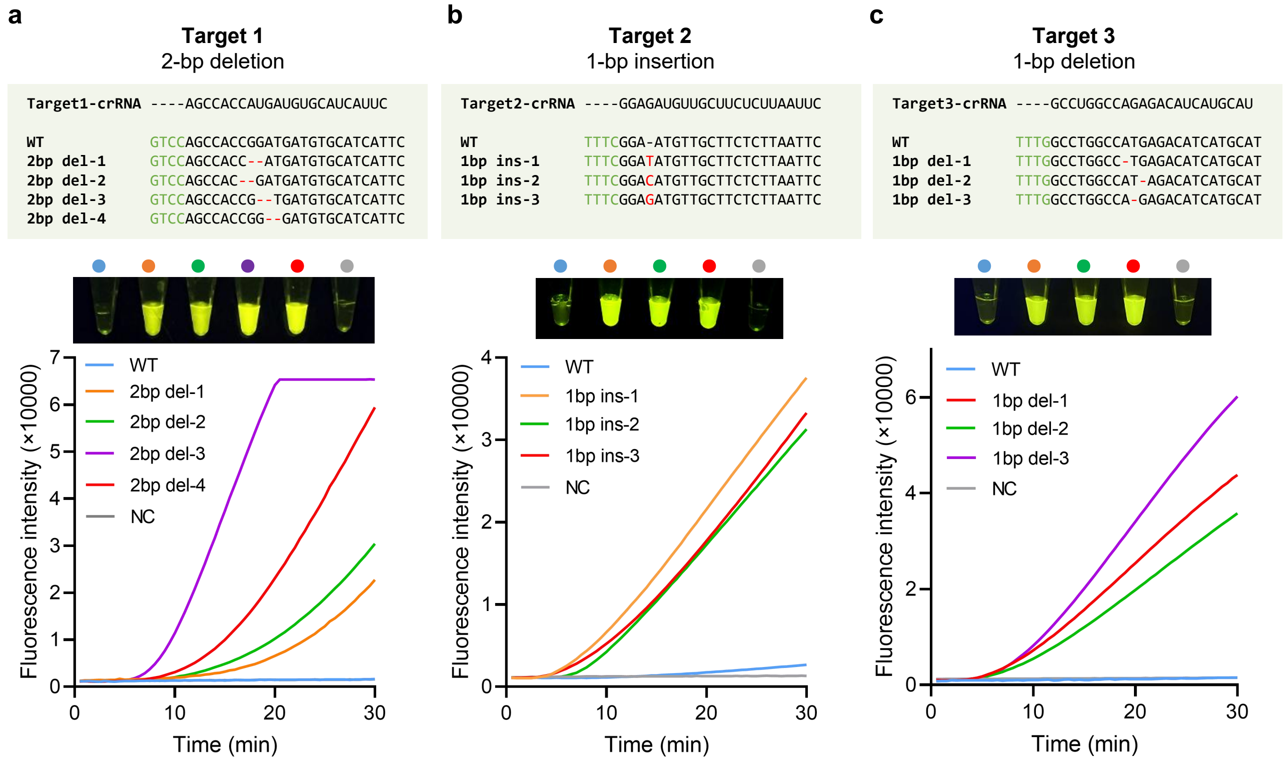

Supplementary Figure 13 | Detect 2-bp and 1-bp indels using the CoHIT system. Sequences of the crRNAs and the DNA templates, naked-eye results, and time course of fluorescence changes for Target 1 (**a**), Target 2 (**b**), and Target 3 (**c**). Indel bases and PAMs in DNA sequences are marked in red and green, respectively. Source data are provided as a Source Data file.

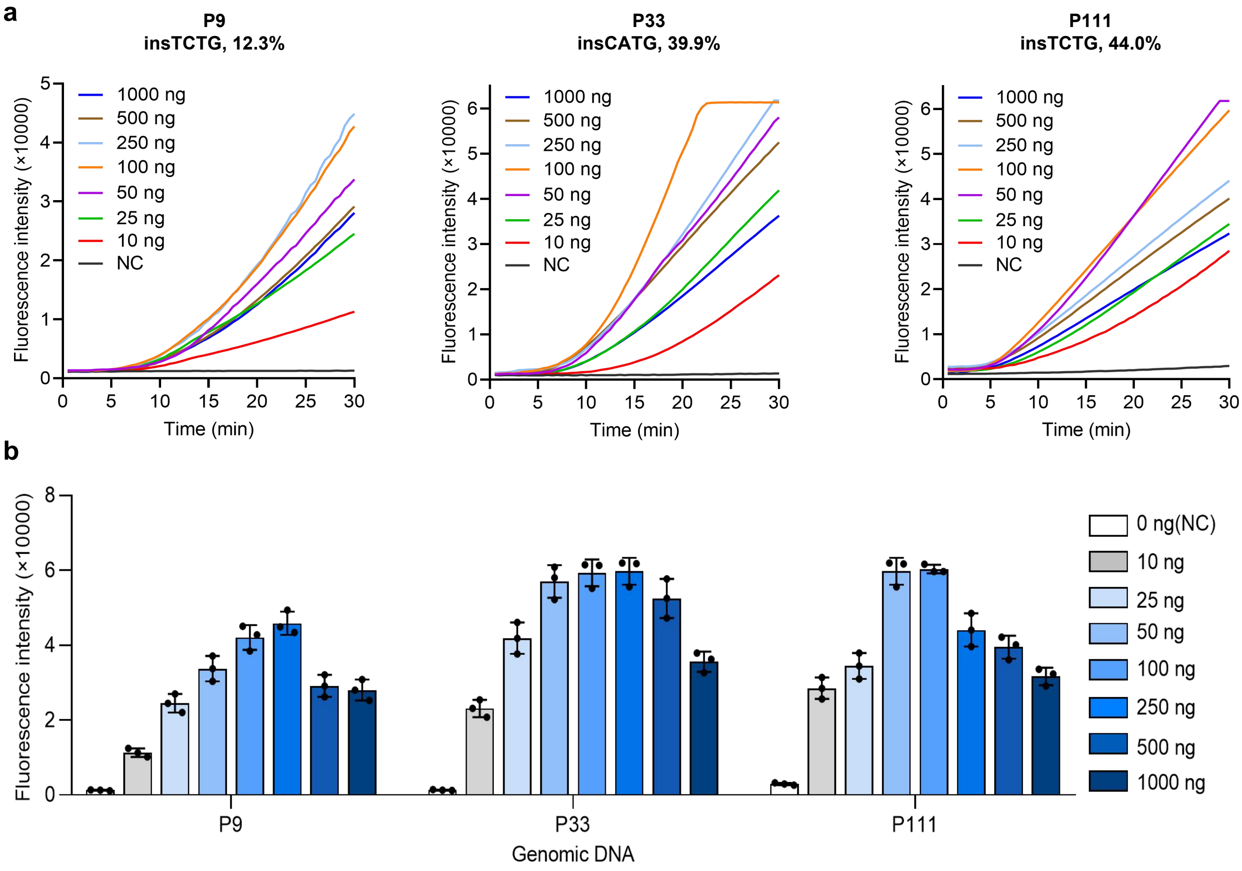

Supplementary Figure 14 | Template input assay of the CoHIT system. a Time course of CoHIT detection using 0~1000 ng genomic DNA of P9, P33, and P111. **b** Statistical chart of the final fluorescence intensities after 30 min of reaction at 39℃. Values and error bars reflect the means and s.d. of three biological replicates. Source data are provided as a Source Data file.

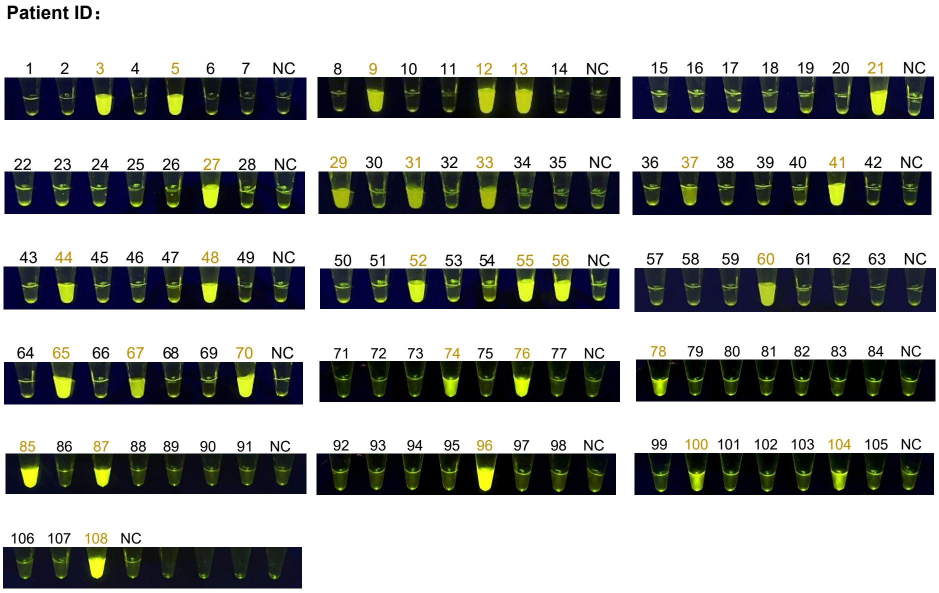

Supplementary Figure 15 | Naked-eye results of CoHIT detection of the *NPM1* gene c.863_864 4-bp insertion status of 108 AML patient samples. Positive samples are marked by yellow ID. Source data are provided as a Source Data file.

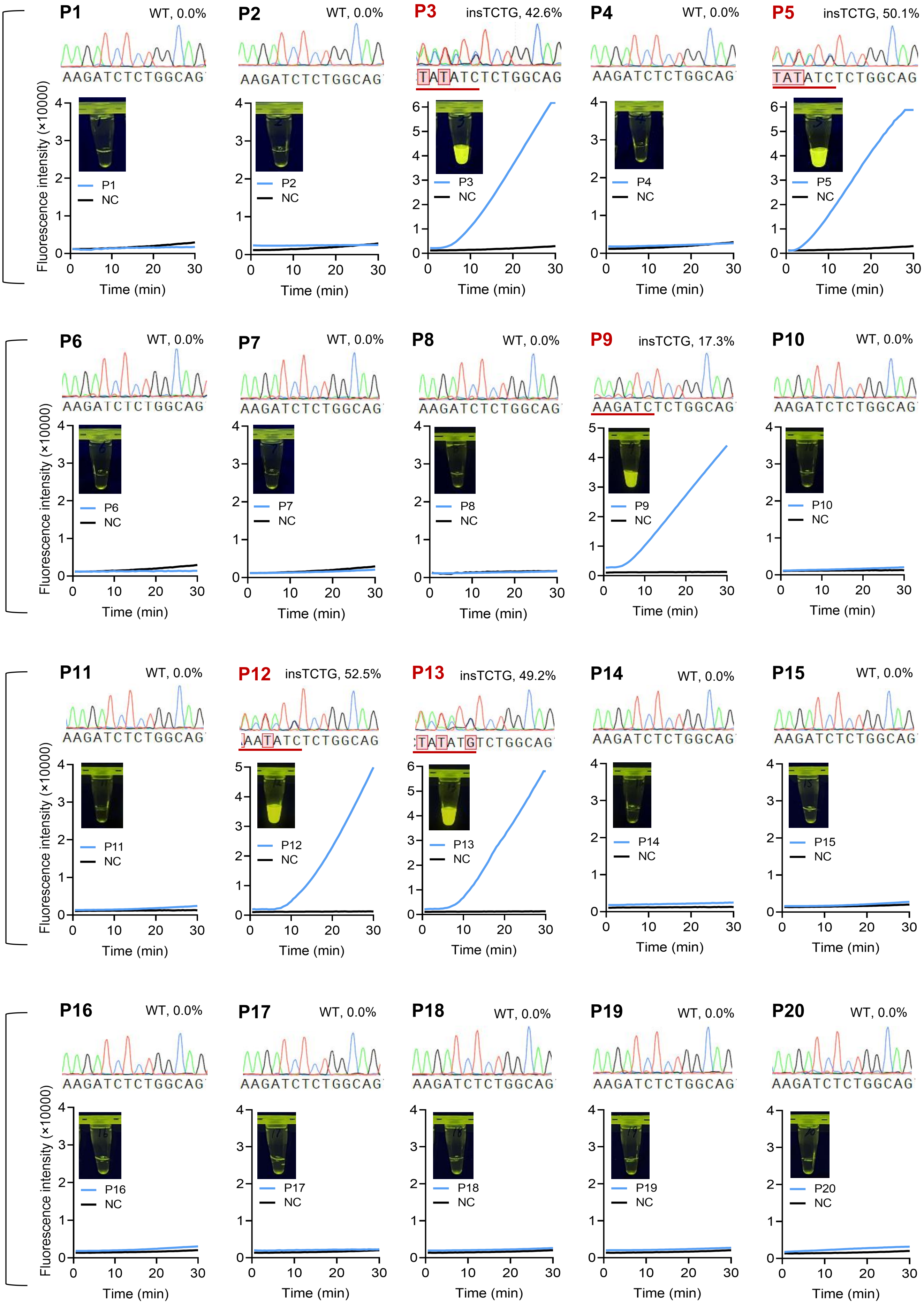

Supplementary Figure 16 | FGS, NGS, and CoHIT detection results of the *NPM1* c.863_864 4-bp insertion status of AML patient samples (P1 ~ 20). FGS results are shown in peaks and mutated bases are underlined in red. Mutation forms and rates of NGS results are shown at the top right corner. Both time-course and naked-eye result of CoHIT detection are shown below. Source data are provided as a Source Data file.

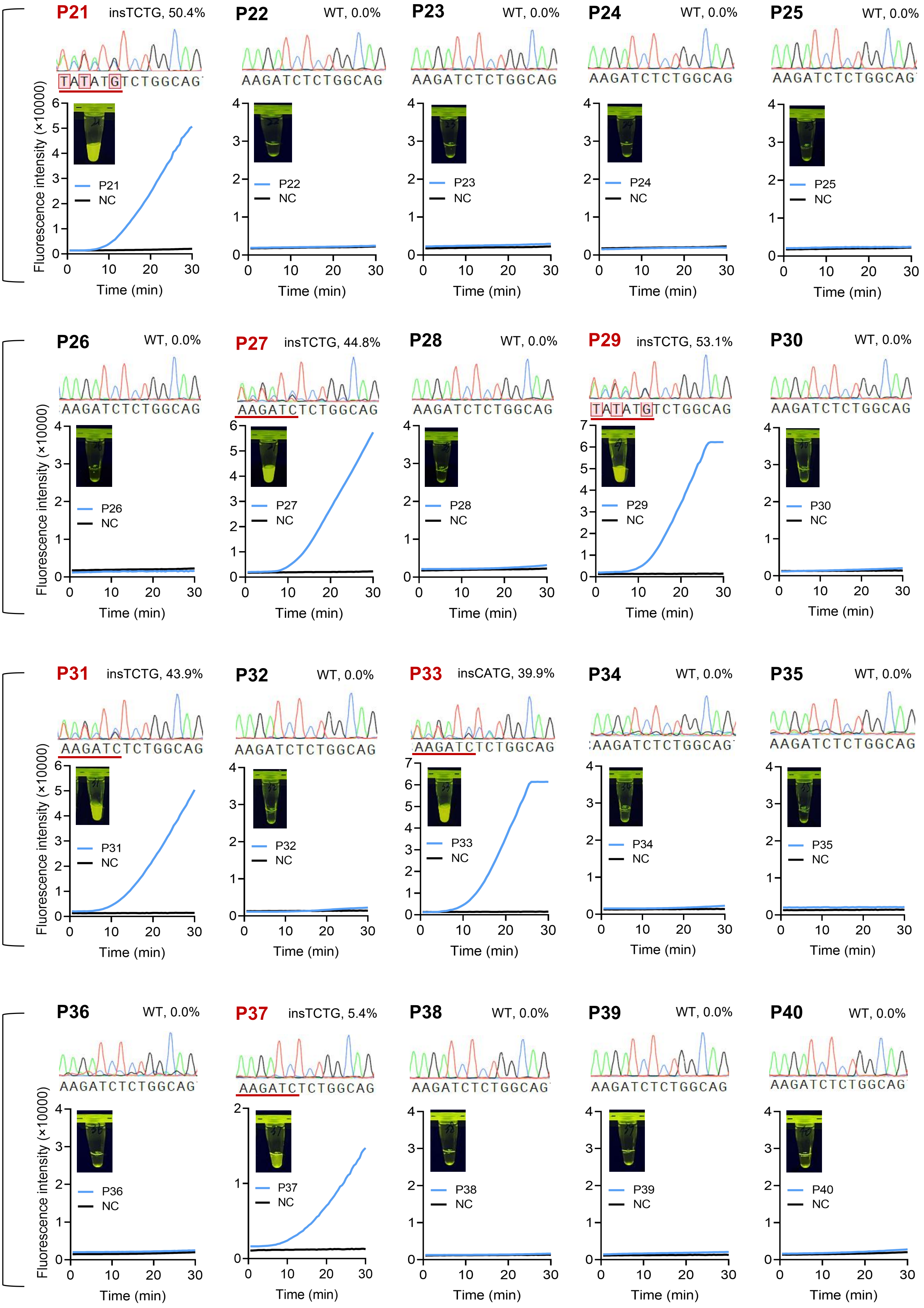

Supplementary Figure 17 | FGS, NGS, and CoHIT detection results of the *NPM1* c.863_864 4-bp insertion status of AML patient samples (P21 ~ 40). FGS results are shown in peaks and mutated bases are underlined in red. Mutation forms and rates of NGS results are shown at the top right corner. Both time-course and naked-eye result of CoHIT detection are shown below. Source data are provided as a Source Data file.

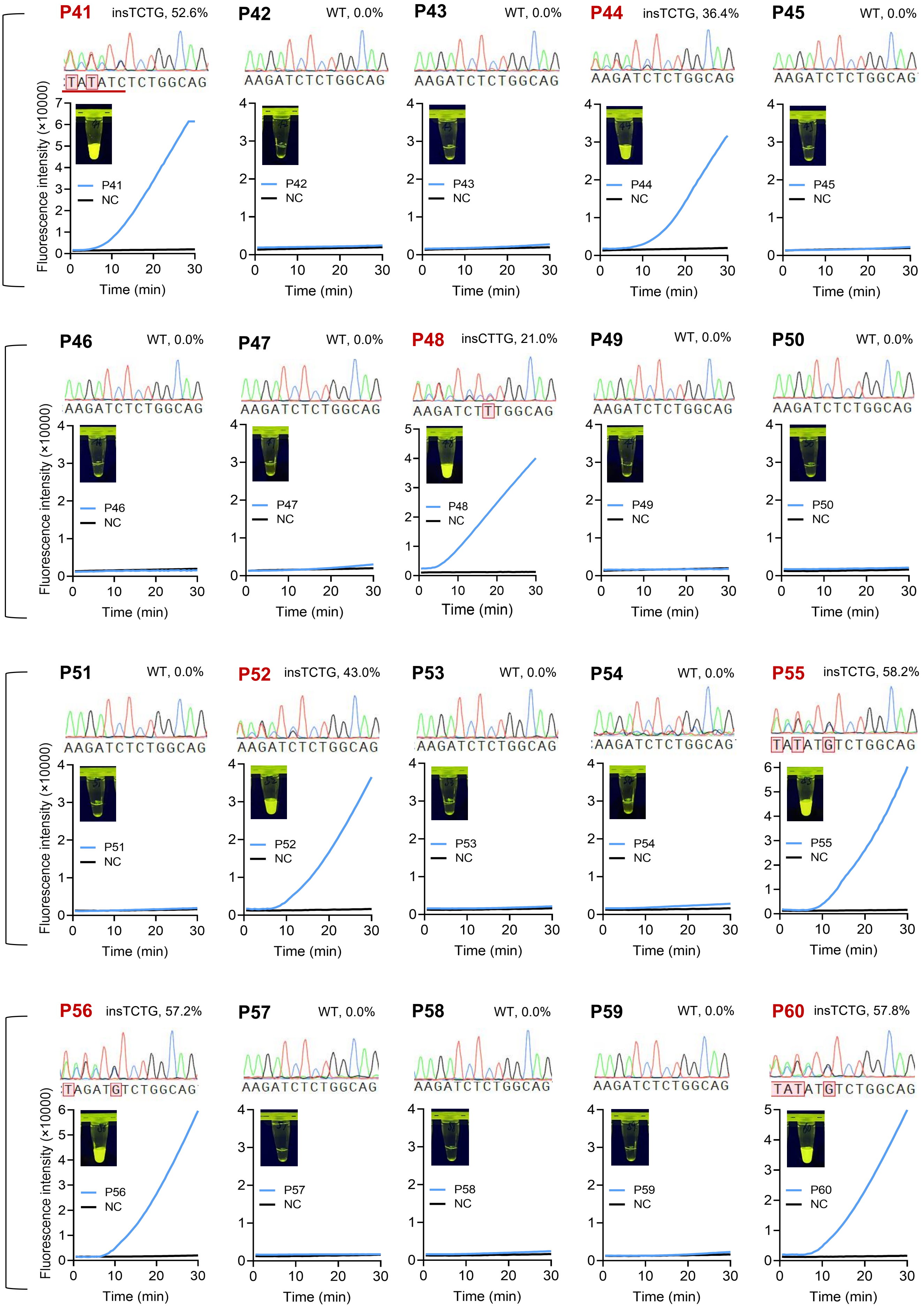

Supplementary Figure 18 | FGS, NGS, and CoHIT detection results of the *NPM1* c.863_864 4-bp insertion status of AML patient samples (P41 ~ 60). FGS results are shown in peaks and mutated bases are underlined in red. Mutation forms and rates of NGS results are shown at the top right corner. Both time-course and naked-eye result of CoHIT detection are shown below. Source data are provided as a Source Data file.

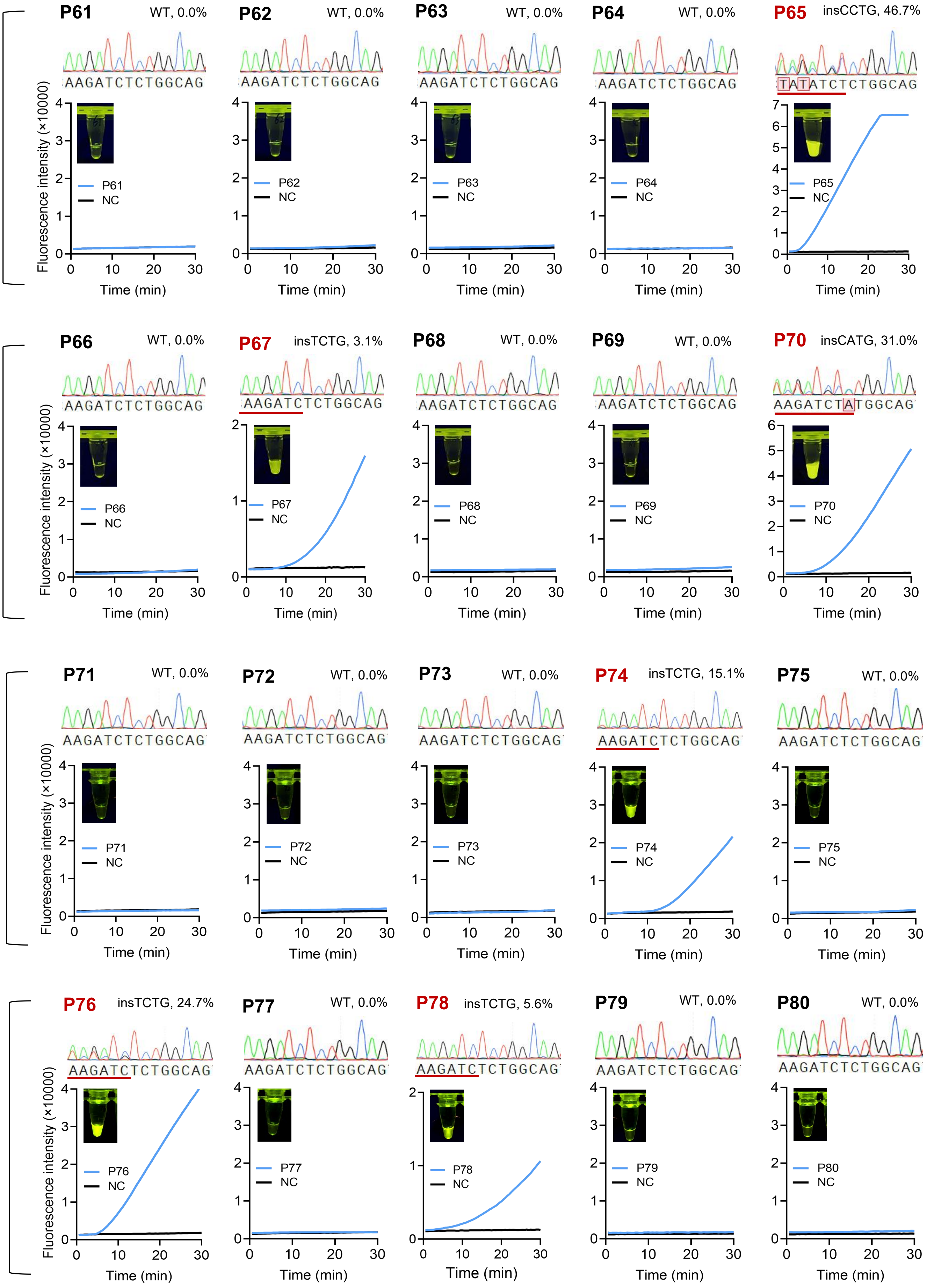

Supplementary Figure 19 | FGS, NGS, and CoHIT detection results of the *NPM1* c.863_864 4-bp insertion status of AML patient samples (P61 ~ 80). FGS results are shown in peaks and mutated bases are underlined in red. Mutation forms and rates of NGS results are shown at the top right corner. Both time-course and naked-eye result of CoHIT detection are shown below. Source data are provided as a Source Data file.

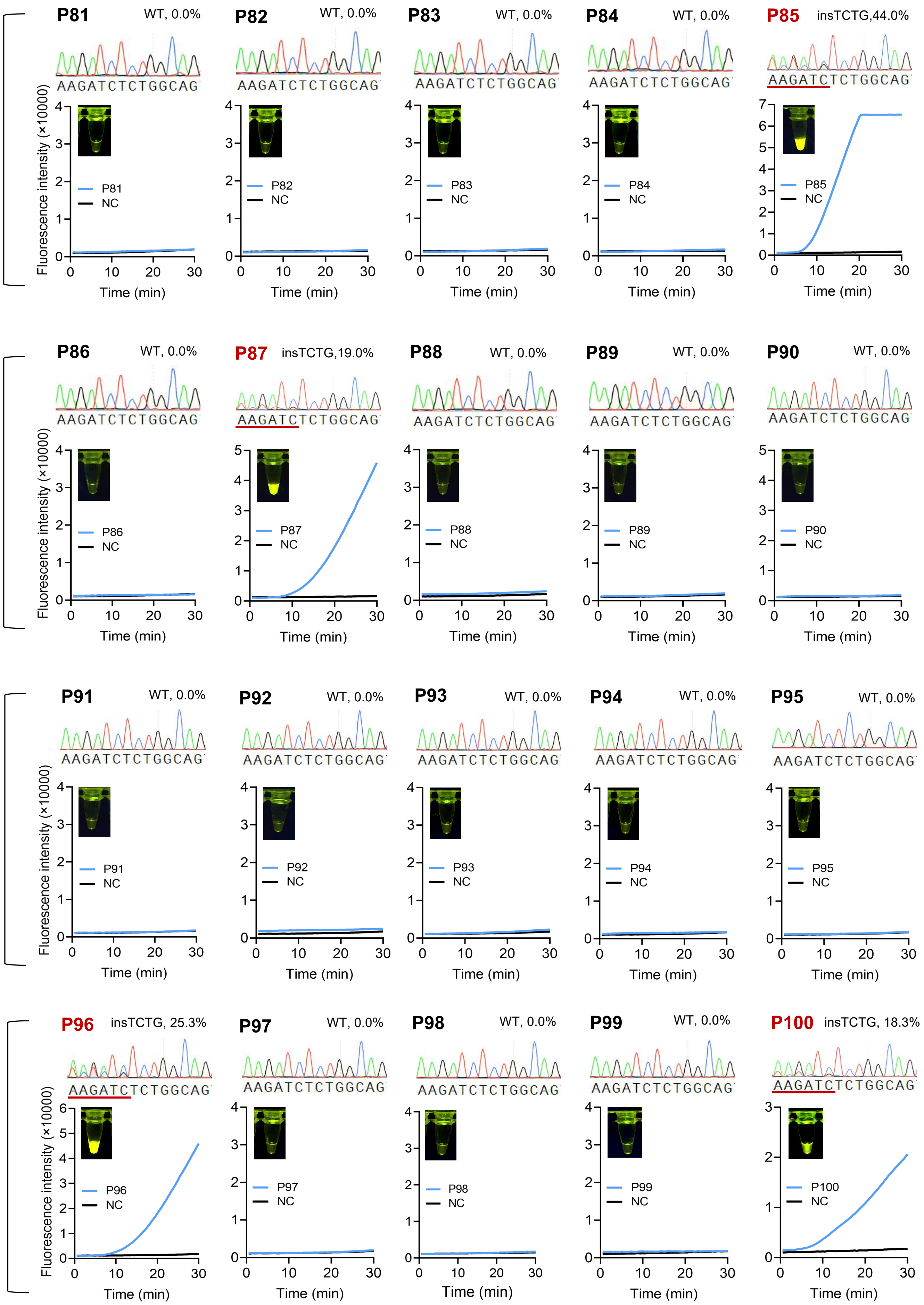

Supplementary Figure 20 | FGS, NGS, and CoHIT detection results of the *NPM1* c.863_864 4-bp insertion status of AML patient samples (P81 ~ 100). FGS results are shown in peaks and mutated bases are underlined in red. Mutation forms and rates of NGS results are shown at the top right corner. Both time-course and naked-eye result of CoHIT detection are shown below. Source data are provided as a Source Data file.

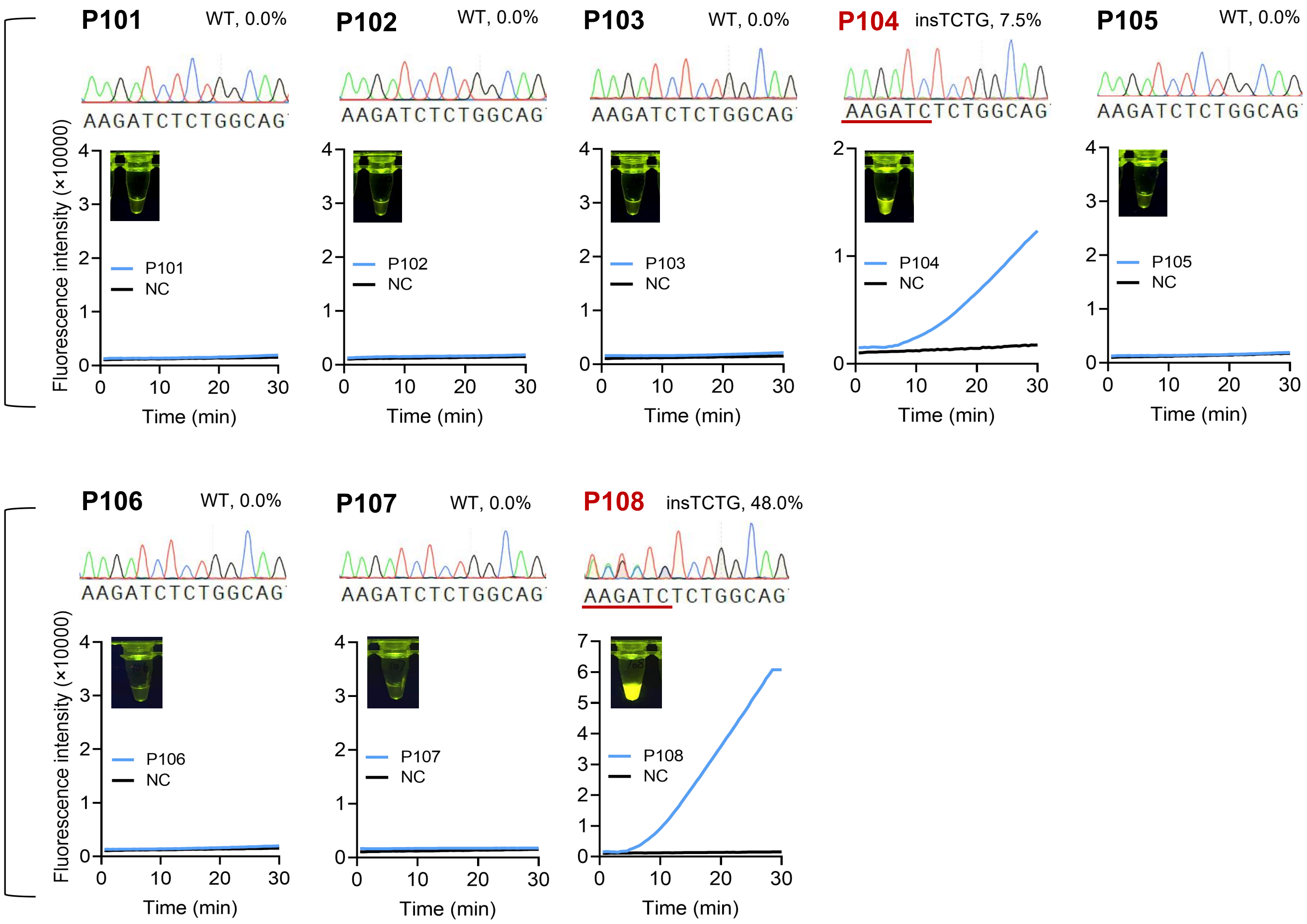

Supplementary Figure 21 | FGS, NGS, and CoHIT detection results of the *NPM1* c.863_864 4-bp insertion status of AML patient samples (P101 ~ 108). FGS results are shown in peaks and mutated bases are underlined in red. Mutation forms and rates of NGS results are shown at the top right corner. Both time-course and naked-eye result of CoHIT detection are shown below. Source data are provided as a Source Data file.

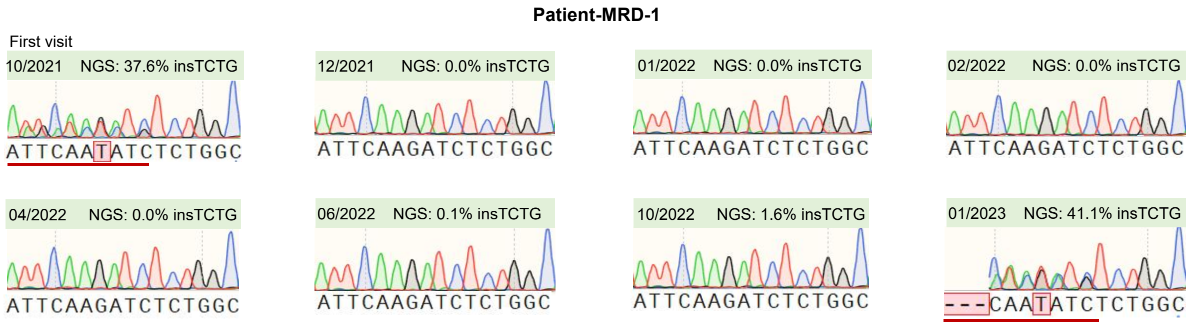

Supplementary Figure 22 | FGS chromatograms of the eight bone marrow samples of Patient-MRD-1, collected between 10/2021 (First visit) and 01/2023. Mutant peaks are underlined in red. The percentages near the dates are NGS results of NPM1 gene c.863_864insTCTG mutation.

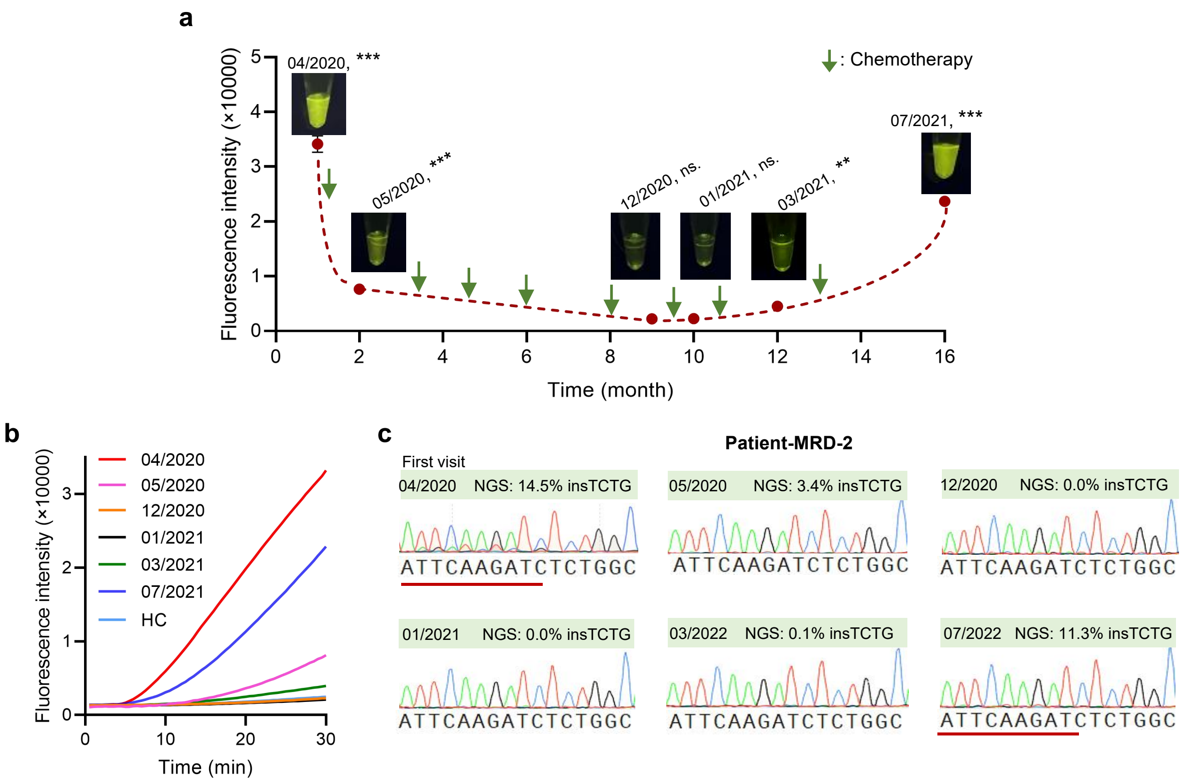

Supplementary Figure 23 | FGS, NGS, and CoHIT detection results of Patient-MRD-2. **a** Six bone marrow samples were collected between 04/2020 (First visit) and 07/2021 and tested by CoHIT assay. Final fluorescence values and naked-eye photos of the six samples are shown in chronological order. The asterisks indicate P values compared with healthy WT control (HC). **b** Time course of CoHIT detection of the six samples and HC. **c** FGS chromatograms of the six samples. Mutant peaks are underlined in red. The percentages near the dates are NGS results of the *NPM1* gene c.863_864insTCTG mutation. Source data are provided as a Source Data file.

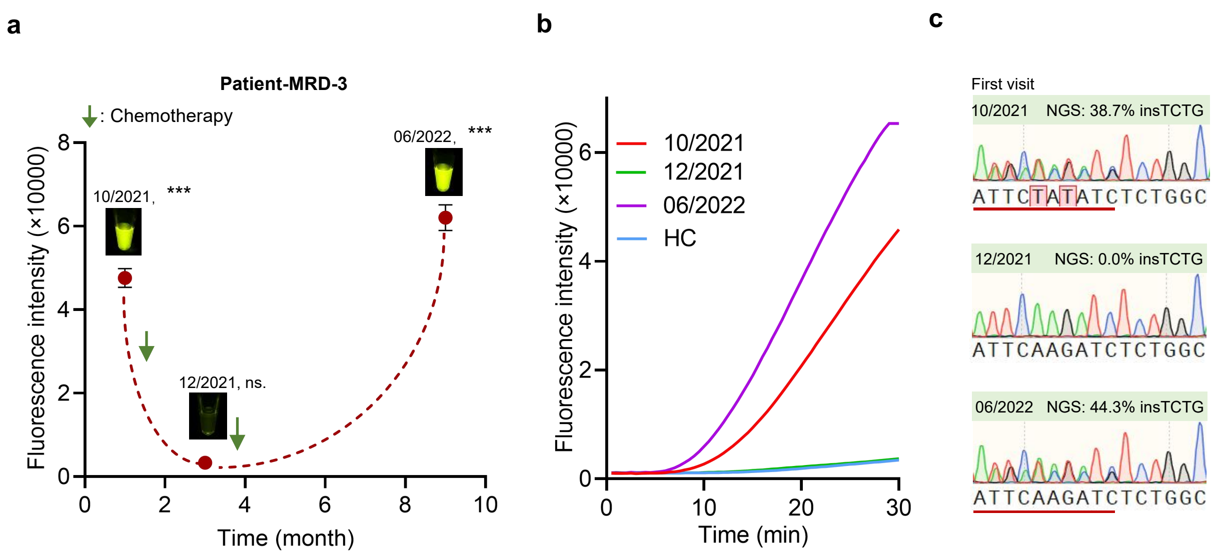

Supplementary Figure 24 | FGS, NGS, and CoHIT detection results of Patient-MRD-3. **a** Three bone marrow samples were collected between 10/2021 (First visit) and 06/2022 and tested by CoHIT assay. Final fluorescence values and naked-eye photos of the three samples were shown in chronological order.The asterisks indicate P values compared with healthy WT control (HC). **b** Time-course of CoHIT detection of the three samples and HC. **c** FGS chromatograms of the three samples. Mutant peaks are underlined in red. The percentages near the dates are NGS results of *NPM1* gene c.863_864insTCTG mutation. Source data are provided as a Source Data file.

Target	Gene mutation
1	*NPM1* c.863_864 4-bp insertion
2	*FLT3* c.1770_1771 24-bp insertion
3	*FLT3* c.1793_1794 21-bp insertion
4	*FLT3* c.1790_1791 18-bp insertion
5	*FLT3* c.1776_1777 18-bp insertion
6	*FLT3* c.1784_1785 21-bp insertion
7	*FLT3* c.1796_1797 27-bp insertion
IC	*GAPDH* WT

Supplementary Figure 25 | Gene mutation sites and types of targets 1 ~ 7 in the microfluidic chip-based multiplexing CoHIT assay.

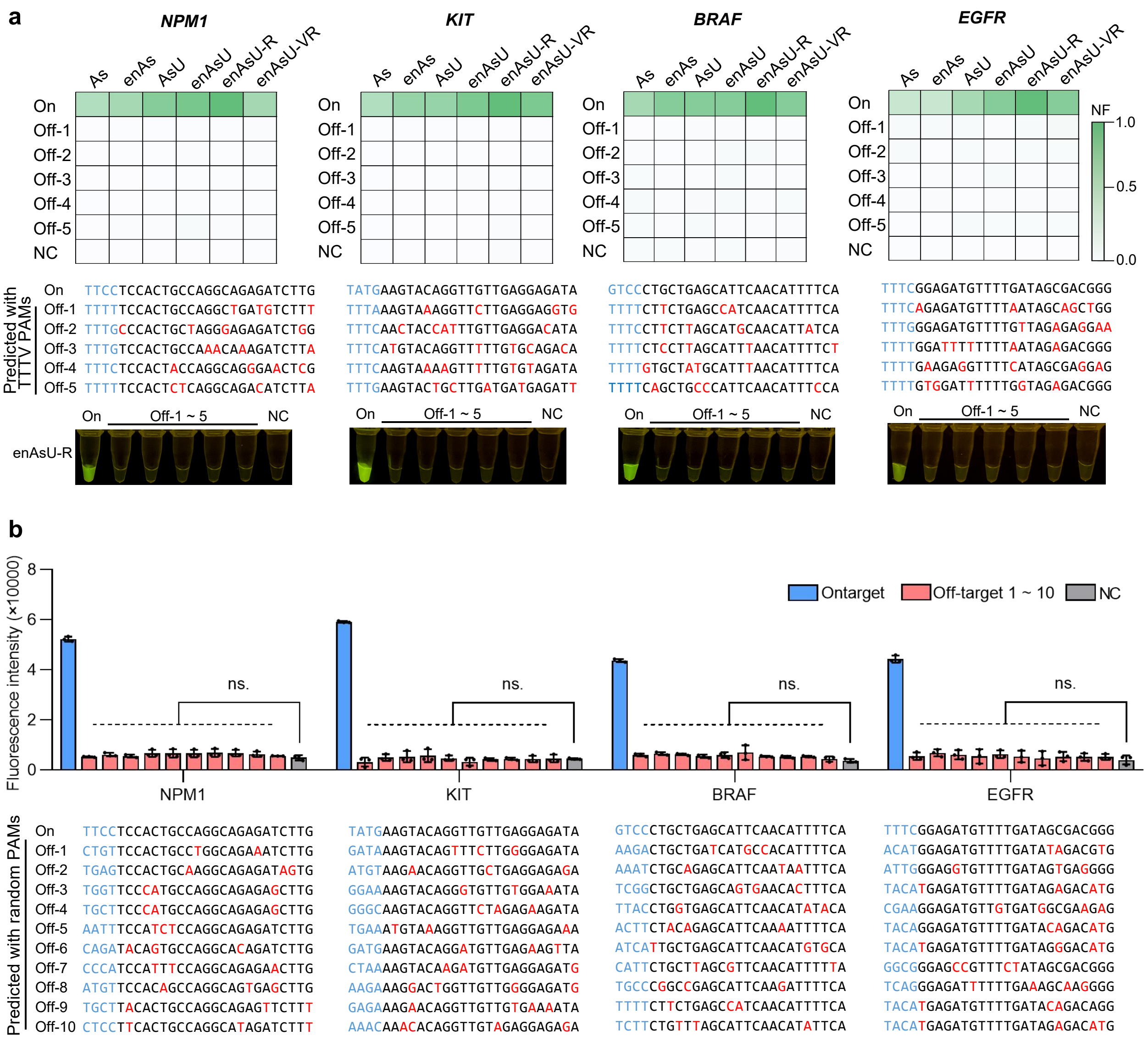

Supplementary Figure 26 | Off-target evaluation of Cas12a proteins on four sites. **a** Detection of top five predicted off-targets in human genome with TTTN PAM using six different Cas12a proteins. PAM and mismatch bases in the sequences are marked in blue and red, respectively. The off-targets were predicted using the online web tool Cas-OFFinder (http://www.rgenome.net/cas-offinder/), then PCR-amplified and purified for the Cas12a in vitro cleavage assay, with 5E10 copies of DNA as substrates, reacting for 30 min. Naked-eye photos show the results of enAsU-R Cas12a. **b** Detection of top ten predicted off-targets in human genome without PAM limitation using enAsU-R Cas12a. Values and error bars reflect the means and s.d. of three biological replicates. P values are determined by two-tailed Student's t-tests, ns., no significance. The off-target sequences are listed in Supplementary Table 3 and 4. Source data are provided as a Source Data file.

Comparison between different genotyping methods

	FGS	NGS	qPCR	ddPCR	CoHIT
Mutation LoD (%)	10 [1]	0.01 [2]	0.01	0.001	**0.01**
Turnaround time	~ 1 d	> 5 d	~ 2 h	4 h [3]	**< 30 min**
Instrument cost	+++	+++	++	+++	**+**
Operational complexity	+++	+++	++	+++	**+**
Result analysis difficulty	+++	+++	++	++	**+**
Cost per sample	+	+++	++	++	**+**

[1] J Med Virol. 2020 Dec;92(12):3604-3608.
[2] Lancet. 2023 Jun 17;401(10393):2073-2086.
[3] Nat Biomed Eng. 2017;1:714-723.
* +, ++, and +++ means low, moderate, and high.

Supplementary Table 1 | Comparison between different genotyping methods.

Supplementary References

1. Akuta N, Suzuki F, Kobayashi M, et al. Detection of TERT promoter mutation in serum cell-free DNA using wild-type blocking PCR combined with Sanger sequencing in hepatocellular carcinoma. J Med Virol. 2020;92(12):3604-3608. doi:10.1002/jmv.25724

2. DiNardo CD, Erba HP, Freeman SD, Wei AH. Acute myeloid leukaemia. Lancet. 2023;401(10393):2073-2086. doi:10.1016/S0140-6736(23)00108-3

3. Wu LR, Chen SX, Wu Y, Patel AA, Zhang DY. Multiplexed enrichment of rare DNA variants via sequence-selective and temperature-robust amplification [published correction appears in Nat Biomed Eng. 2017 Dec;1(12):1005]. Nat Biomed Eng. 2017;1:714-723. doi:10.1038/s41551-017-0126-5
