# Supplementary material for: CoHIT: a one-pot ultrasensitive ERA-CRISPR system for detecting multiple same-site indels
Source: Nat Commun. 2024 Jun 12;15:5014. doi: 10.1038/s41467-024-49414-7 (PMC11169540; doi:10.1038/s41467-024-49414-7)
Supplement: Supplementary file 3 — Description of Additional Supplementary Files [file 41467_2024_49414_MOESM3_ESM.pdf]

Description of Additional Supplementary Files

Supplementary Data 1. Mutation classification of 2348 patients with NPM1 gene c.863_864 4-bp insertions.

Supplementary Data 2. Sequences of the 32 random TA clones with NPM1 mutations

Supplementary Data 3. Top 5 potential off-targets predicted by Cas-OFFinder with canonical Cas12a TTTN PAM

Supplementary Data 4. Top 10 genome-wide potential off-targets predicted without canonical Cas12a TTTN PAM

Supplementary Data 5. dsDNA fragments used in mismatch tolerance assay and 2/1-bp small indels detection

Supplementary Data 6. PCR primer sequences

Supplementary Data 7. Amino acid sequences of Cas12a proteins used in this study

Supplementary Data 8. crRNA sequences

Supplementary Data 9. ERA primer sequences

Supplementary Data 10. Next-generation sequencing primer sequences
