# Supplementary material for: CoHIT: a one-pot ultrasensitive ERA-CRISPR system for detecting multiple same-site indels
Source: Nat Commun. 2024 Jun 12;15:5014. doi: 10.1038/s41467-024-49414-7 (PMC11169540; doi:10.1038/s41467-024-49414-7)
Supplement: Supplementary file 5 — Reporting Summary [file 41467_2024_49414_MOESM5_ESM.pdf]

nature portfolio

Corresponding author(s): Kui Li, Xinjie Wang, Fuling Zhou

Last updated by author(s): May 25, 2023

Reporting Summary

Nature Portfolio wishes to improve the reproducibility of the work that we publish. This form provides structure for consistency and transparency in reporting. For further information on Nature Portfolio policies, see our Editorial Policies and the Editorial Policy Checklist.

Statistics

For all statistical analyses, confirm that the following items are present in the figure legend, table legend, main text, or Methods section.

n/a	Confirmed	
☐	☒	The exact sample size (*n*) for each experimental group/condition, given as a discrete number and unit of measurement
☐	☒	A statement on whether measurements were taken from distinct samples or whether the same sample was measured repeatedly
☐	☒	The statistical test(s) used AND whether they are one- or two-sided<br>*Only common tests should be described solely by name; describe more complex techniques in the Methods section.*
☒	☐	A description of all covariates tested
☒	☐	A description of any assumptions or corrections, such as tests of normality and adjustment for multiple comparisons
☐	☒	A full description of the statistical parameters including central tendency (e.g. means) or other basic estimates (e.g. regression coefficient) AND variation (e.g. standard deviation) or associated estimates of uncertainty (e.g. confidence intervals)
☐	☒	For null hypothesis testing, the test statistic (e.g. *F*, *t*, *r*) with confidence intervals, effect sizes, degrees of freedom and *P* value noted<br>*Give P values as exact values whenever suitable.*
☒	☐	For Bayesian analysis, information on the choice of priors and Markov chain Monte Carlo settings
☒	☐	For hierarchical and complex designs, identification of the appropriate level for tests and full reporting of outcomes
☒	☐	Estimates of effect sizes (e.g. Cohen's *d*, Pearson's *r*), indicating how they were calculated

Our web collection on statistics for biologists contains articles on many of the points above.

Software and code

Policy information about availability of computer code

Data collection	Illumina NextSeq 500 (2 × 150) platform
Data analysis	Statistical analyses were carried out with GraphPad Prism 8.0 (GraphPad Software, CA, USA) and SPSS 27 (IBM, Armonk, NY, USA).<br>For deep sequencing analysis, the sequencing reads were demultiplexed using AdapterRemoval (version 2.2.2). All processed reads were then mapped to the target sequences using the BWA-MEM algorithm (BWA v0.7.17). For targeted indel mutations, mutation efficiency was calculated as: number of indel-containing reads/total mapped reads (%). For PAM analysis, cleavage efficiency was calculated as: 100%-[Cleaved(specific PAM reads/inner uncleavable control reads)/Initial(specific PAM reads/inner uncleavable control reads)]<br>Students' t-test (two-tailed) were used to determine the statistical significance of differences in levels among experimental groups. Quantitative data are expressed as the mean value ± standard error. All the statistical details of the experiments can be found in the figure legends. No statistical method was used to predetermine the sample size. No data were excluded from any of the experiments.

For manuscripts utilizing custom algorithms or software that are central to the research but not yet described in published literature, software must be made available to editors and reviewers. We strongly encourage code deposition in a community repository (e.g. GitHub). See the Nature Portfolio guidelines for submitting code & software for further information.

Data

Policy information about availability of data

All manuscripts must include a data availability statement. This statement should provide the following information, where applicable:

- Accession codes, unique identifiers, or web links for publicly available datasets
- A description of any restrictions on data availability
- For clinical datasets or third party data, please ensure that the statement adheres to our policy

The authors declare that all data of this study are available within the article and its supplementary files. NGS data has been deposited to the NCBI-SRA repository under BioProject number: PRJNA1029775 (https://www.ncbi.nlm.nih.gov/bioproject/?term=PRJNA1029775). The reference human genome assembly GRCh38/hg38 used for reads mapping is an openly accessible resource (https://www.ncbi.nlm.nih.gov/assembly/GCF_000001405.40). Source data are provided with this paper. Other data and details are available from the corresponding authors upon reasonable request.

Research involving human participants, their data, or biological material

Policy information about studies with human participants or human data. See also policy information about sex, gender (identity/presentation), and sexual orientation and race, ethnicity and racism.

Reporting on sex and gender	Not applicable. Population grouping is not involved in this study. No sex or gender analysis was carried out. All patient samples in this study were deidentified and patient demographics are not available.
Reporting on race, ethnicity, or other socially relevant groupings	Not applicable. Population grouping is not involved in this study. All patient samples in this study were deidentified and patient demographics are not available.
Population characteristics	A total of 125 blood samples of adult patients with acute myeloid leukemia were randomly collected from the Hematology Department in Zhongnan Hospital of Wuhan University under an approved Institutional Review Board protocol. These patients are with acute myeloid leukemia. The information of their names, gender, age, and race are hidden in this research.
Recruitment	The patients with acute myeloid leukemia were randomly selected.
Ethics oversight	Blood samples in this study were collected from the Haematology Department of Zhongnan Hospital of Wuhan University under an approved Institutional Review Board protocol (Scientific Ethics Approval No. 2017064). For all human patient samples, informed written consent was obtained prior to donation. Each participant received nutritional products as compensation for their participation.

Note that full information on the approval of the study protocol must also be provided in the manuscript.

Field-specific reporting

Please select the one below that is the best fit for your research. If you are not sure, read the appropriate sections before making your selection.

☒ Life sciences ☐ Behavioural & social sciences ☐ Ecological, evolutionary & environmental sciences

For a reference copy of the document with all sections, see nature.com/documents/nr-reporting-summary-flat.pdf

Life sciences study design

All studies must disclose on these points even when the disclosure is negative.

Sample size	Samples sizes were indicated in figure legends. We did not predetermine sample sizes. In this study, we detected 125 blood samples of adult patients with acute myeloid leukemia.
Data exclusions	No data has been excluded from the analyses.
Replication	As indicated in the methods, all data represented are results from biological replicates. The average values from such replications are presented (+/- STD, as indicated in the legend) . In addition, the conclusions are made on analyses at multiple target sites.
Randomization	The patients with acute myeloid leukemia were randomly selected.
Blinding	This study did not involve human grouping. When collecting the patient blood samples, we did not know their NPM1 mutation status. The FGS, NGS, and CoHIT detection were taken at the same time for genotyping and comparison.

Reporting for specific materials, systems and methods

We require information from authors about some types of materials, experimental systems and methods used in many studies. Here, indicate whether each material, system or method listed is relevant to your study. If you are not sure if a list item applies to your research, read the appropriate section before selecting a response.

Materials & experimental systems

n/a	Involved in the study
☒	☐ Antibodies
☒	☐ Eukaryotic cell lines
☒	☐ Palaeontology and archaeology
☒	☐ Animals and other organisms
☒	☐ Clinical data
☒	☐ Dual use research of concern
☒	☐ Plants

Methods

n/a	Involved in the study
☒	☐ ChIP-seq
☒	☐ Flow cytometry
☒	☐ MRI-based neuroimaging
